# Medical and Physical Intelligence

**Published:** 1822-04

**Authors:** 


### Medical and Physical Intelligence.

### 1. *On Sulphureous Mineral Waters, and the Nitrogen contained in them.*

THE existence of nitrogen in mineral waters has frequently been ascertained, and its quality stated ; but no very precise ideas have hitherto been published on the cause of its existence in the waters, and the attempt of M. Anglada is, perhaps, the first made to show its source, and the situations in which it may be expected. Having observed, from the experience of others and himself, that nitrogen occurred in waters containing sulphuretted hydrogen, he was induced to search whether it was not constantly present in such waters ; and, finding that to be the case, reasoned and experimented upon its production. In many sulphureous springs, the sources of which were easily arrived at, it was readily observed, that nitrogen either rose from the sides of the spring, or could be obtained without difficulty from the water ; but in others, which were confined by pipes or conduits, it was necessary to open up the works, and get to the source. In all cases, however, sulphureous waters were found to contain nitrogen ; and it was remarked that, though the water contained abundance of sulphuretted hydrogen, the gas contained none.

M. Anglada expected, by heat, to obtain sulphuretted hydrogen and carbonic acid gas from sulphureous water ; but, in no case, obtained any thing except pure nitrogen gas. Guided by this result, he concluded that, in all the sulphureous waters that had thus yielded him nitrogen, that gas came from the air which the waters had taken up in their subterraneous course, and from which the oxygen had been separated by the sulphur, &c. In order, as it were, to prove this opinion, a portion of a sulphureous water was treated with acetate of lead, to separate all the sulphuretted hydrogen from it, and then boiled : it gave out more gas than before, and the gas was a mixture of oxygen and hydrogen.

The air, thus furnished to waters, is given to them, M. Anglada thinks, in the bowels of the earth, by currents of air, of which we know little or nothing.

In consequence of this action of the air on waters of this kind, it happens sometimes that a water, decidedly sulphureous at its source, ceases to be so at a little distance from it; dependant on the strength of the water, and the means it has of getting air. In those cases, the previous sulphureous state of the water may be deduced from the disengagement of pure nitrogen, or containing very little oxygen, and from certain glairy appearances which are exhibited by those waters.

M. Anglada also remarks, that, in many waters containing carbonic acid, abundance of nitrogen is found ; and he suggests that, probably, the air may act in them on some carbonaceous matters, giving rise to carbonic acid and nitrogen at the same time. It is also remarked, in the conclusions attached to this paper, (and which, being given above, need not be repeated,) that the change takes place at all temperatures; and that, if nitrogen is not found in every sulphureous water, it is in all those containing a hydro-sulphuretted alkali.*—*Ann. de Chim.*, vol. xviii. p. 113.

### 2. *First Appearance of the Boa Constrictor in the Island of St. Vincent.*

A most singular circumstance occurred, last week, in the Charaib country, when some negroes, who were working near Sandy Bay, discovered an immense serpent, hitherto wholly unknown in any of these islands, and which was shot through the head by one of the party. It is supposed to be a species of *boa*, so common on the neighbouring continent; but in what way it reached the shores of St. Vincent, is quite unknown. Its entire length was between fourteen and fifteen feet, the circumference of the body between three and four feet. When first seen it was lying in a coil, but raised itself on being roused.—*Royal Gazette and Bahama Advertiser*, August 1821.

### 3. *Cretinism in a small District of the Kingdom of Würtemberg.*

In the valleys of Kocher and Roth, where there is abundance of stagnant water, there are upwards of 160 cretins, in a comparatively small population. One-third of these are quite deaf. About 300 adults, either male or female, are afflicted with bronchocele. It is worthy of note, that the inflammatory and exanthematic diseases, to which the inhabitants of this district are frequently subject, are seldom, if ever, accompanied with fever, and that no intermittent fever has ever been known to prevail among them. Cretinism, however, seems, of late years, to be on the decrease in these valleys, owing, probably, to the fostering care of government, who have endeavoured to ameliorate the lot of these wretched inhabitants.—*Allgem. Mediz. Zeitung.*

### 4. *New Remedy in Intermittent Fevers.*

Dr. W. Follickoffer, of Baltimore, is preparing for the press a Treatise on the Use of Prussiate of Iron in Intermittent Fevers.

### 5. *Structure of the Chrystalline Lens.*

Dr. Edward G. Howell, of Philadelphia, has ascertained, to his own satisfaction, from researches made in February and March, 1821, that the capsule of the crystalline lens is a distinct membrane, and of the serous kind; demonstrated to be so, from analogy, from its habitudes with certain chemical re-agents, from morbid derangement of structure, &c. He intends, at some future period, to enter more fully into the anatomical structure of the eye, which, so far as information can be obtained from books, is, he thinks, but imperfectly understood.

---

* See an original paper, by the Editor, in the last Number.

### 6. *Vaccine Board.*

A return of the establishment and expences of the National Vaccine Board, stating the name, office, and salary of each person, the place of residence, also the amount of contingent expences, for the year ending 5th January, 1822:—

### *Members of the Board, viz.*

| | £ | s. | d. |
|---|---|---|---|
| Sir Henry Halford, Bart. President of the College of Physicians, Curzon-street | 100 | 0 | 0 |
| Dr. Algernon Frampton, Censor of the College of Physicians, New Broad-street | 100 | 0 | 0 |
| Dr. Thomas Hume, ditto, South-street, Grosvenor-square | 100 | 0 | 0 |
| Dr. Charles Badham, ditto, Berkeley square | 100 | 0 | 0 |
| Dr. Robert Lloyd, ditto, Grosvenor-street | 100 | 0 | 0 |
| Sir Everard Home, Bart. President of the College of Surgeons, Sackville-street | 100 | 0 | 0 |
| Sir William Blizard, Knt. Devonshire-square, } Vice-Presidents of ditto | 100 | 0 | 0 |
| Henry Cline, Esq. Lincoln's-Inn Fields } Vice-Presidents of ditto | 100 | 0 | 0 |
| *Director*—James Moore, Esq. Conduit-street | 200 | 0 | 0 |
| *Registrar*—Dr. James Hervey, No. 18, Percy-street | 300 | 0 | 0 |
| *Secretary*—C. Murray, Esq. 13, John street, Bedford-row | 50 | 0 | 0 |

### *Stationary Vaccinators.*

| | £ | s. | d. |
|---|---|---|---|
| Mr. J. C. Carpue, principal station, 18, Percy-street | 150 | 0 | 0 |
| Mr. J. Addington, 5, Raven-row, Spitalfields | 100 | 0 | 0 |
| Mr. C. Aikin, 4, Broad-street Buildings | 100 | 0 | 0 |
| Mr. J. A. Gillham, school-room, near Surrey Chapel, Blackfriars-Road | 100 | 0 | 0 |
| Mr. Edw. Leese, Gloucester-place | 100 | 0 | 0 |
| Mr. Sawrey, 27, Bedford-row | 100 | 0 | 0 |
| Mr. Fred. Ager, 141, High-street, Whitechapel | 50 | 0 | 0 |
| Mr. J. Barnett, St. John-street, West Smithfield | 50 | 0 | 0 |
| Mr. Jos. Freeman, 20, Spring Gardens | 50 | 0 | 0 |
| Mr. J. P. Peregrine, 3, Halfmoon-street | 50 | 0 | 0 |
| Mr. J. H. Sterry, Alscot-place, Grange-road, Bermondsey | 50 | 0 | 0 |
| Mr. Jos. Ward, Wellclose-square | 50 | 0 | 0 |
| Rent of Furnished House, No. 18, Percy-street | 260 | 0 | 0 |
| Housekeeper | 40 | 0 | 0 |
| Messenger, 1*l.* per week | 52 | 0 | 0 |
| To E. Williams, stationer, 11, Stand | 21 | 12 | 3 |
| — J. C. Parker, for squares of glass, Spur-street, Leicester-fields | 24 | 10 | 3 |
| — J. Thompson, for ivory points, Great Windmill-street | 42 | 0 | 0 |
| — J. Brettell, for printing | 22 | 11 | 6 |
| — R. Hattersley, for platina vaccinators | 4 | 2 | 4 |
| — F. Pastorelli, for glass tubes, Cross-street, Hatton-garden | 0 | 10 | 6 |
| — W. Winfield, for coals, Hungerford Wharf, Strand | 25 | 10 | 6 |
| — Sundry small sums in Registrar's petty cash-book | 17 | 10 | 8 |
| — Audit dinner, 4th July, 1821 ... 17*l.* 3*s.* 0*d* }<br>Deduct fines paid by the members ... 7 2 6 } | 10 | 0 | 6 |
| — Postchaise for Senior Censor and Director, to visit some cases at Cheam School, Surrey | 1 | 15 | 6 |
| — Stamps for payment of salaries | 2 | 16 | 0 |
| | £2,825 | 0 | 0 |

| | £ | s. | d. |
|---|---|---|---|
| Brought foward ........ | 2,825 | 0 | 0 |
| December 10, 1821.—Mr. J. Ring, of Charlotte-street, Rathbone-place, was appointed to a station, Castle-street, Oxford-market, held by the late Mr. Ring—Salary 50*l.*; rent of station 25*l.* .................................... | 75 | 0 | 0 |
| | £2,900 | 0 | 0 |

GEO. GILB. CURREY, M. D. F. R. S.
*Treasurer of the College of Physicians, and to the National Vaccine Board.*

*February* 19, 1822;

40, *Halfmoon-street, Piccadilly.*

### 7. *St. Andrew's and Aberdeen Diplomas.*

"*Unocuique suum,*" &c.; or, as the proverb has it, "Give the devil his due."—Cheap and contemptible as the diplomas, granted by the above universities have always been held by the profession, there is a circumstance connected with the manner of bestowing them, which reflects credit on the Senatus of those learned corporations, and which should be more generally known. That the ASPIRANT to the "*honores summi*" of Doctor of Medicine, from either St. Andrew's or Aberdeen, after having procured a common-place certificate from two physicians, has only to forward that document to one or other of those two universities, with the necessary amount for fees, in order to obtain the desired distinction, is a fact with which all those who have obtained the said diplomas are well acquainted; and, among others, Doctors Solomon, Brodum senior, Bell, &c. &c. But the public are, perhaps, not equally aware that such is the scrupulosity with which the senatus of either university discharge their duty on those occasions, that an inquiry is invariably made into the respectability of the signing physicians. The mode of investigation which they pursue is equally simple and ingenious. Suppose H. and D. have signed the certificate; the secretary or registrar writes to H., desiring him to state what he knows of D.; while an application is, at the same time, made to D. for information respecting the character of H. The knowledge of the respectability of the candidate, as well as of his signing friends, thus obtained, is deemed quite effectual to prevent imposition; and the public, in fact, may satisfy themselves of the small number of cases of imposition that have occurred in the case of diplomas granted by either of the universities in question, if they will take the trouble of looking into the list of the M. D.'s who owe their academical honours to those learned bodies. The above fact was, until lately, quite new to us; and we confess that the knowledge of it has had the effect of making us look upon a doctor from St. Andrew's or Marischal College with more respect than we had hitherto been inclined to do: for, although it be true, that these every-day graduates get their honours at an infinitely easier rate than the regular physician of any respectable and well-known university or college, it does appear that they do not succeed in it without the trouble of some *complicated epistolary correspondence.*

8. *Spontaneous Explosion of Chlorine and Hydrogen.*

It has been long known that a mixture of chlorine and hydrogen explodes when exposed to the direct action of the sun's rays. In order to try if this effect could be produced by the radiation of a common culinary fire, Professor Silliman filled a common Florence oil-flask (well cleaned;) half full of chlorine gas, and was in the act of introducing the hydrogen in the pneumatic cistern. "There was not only no *direct* emanation from the sun, but even the *diffuse* light was rendered much feebler than common by a thick snow-storm, which had covered the skylight above with a thick mantle, and veiled the heavens in a singular degree for such a storm. Under these circumstances, the hydrogen was scarcely all introduced before the flask exploded with a distinct flame; portions of the glass stuck in the woodwork of the ceiling of the room, and the face and eyes escaped by being out of the direction of the explosion; nothing but the neck of the flask remained in hand. This occurrence then proves, that a mixture of chlorine and hydrogen gas may explode spontaneously even in a diffuse light, and even in a very dim light."—*American Journal of Science*, Vol. III. No. 2. p. 343.

9. *Heat produced in the Skin by Chlorine.*

Dr. Hare of Philadelphia has found that, when the temperature of the air is about 60°, the hand, when immersed in chlorine, experiences a sensation of heat equal to 90° or 100°, even though the common thermometer should not be affected when immersed. Dr. Hare conjectures, "that a sort of chemical action may take place between the gas and the insensible perspiration of the skin, as the power of chlorine in dissolving animal effluvia is well known."—*American Journal of Science*, Vol. III. No. 2. p. 344.

10. *Camphor.*

"In the last number of the Philosophical Journal, you did me the honour to insert some experiments of mine on the solubility of phosphorous in sulphuret of carbon. Permit me now to add, that if a drop of the sulphuret be brought in contact with a chip of camphor while moving on water, the rotatory motion is instantly checked, and a film of camphor diffuses round the spot to some distance. I have sometimes observed, when a small portion of floating sulphuret of carbon is touched by a minute fragment of camphor, that it glances off with extreme rapidity, and is speedily lost in a rotatory circle. If the camphor, when dropped on the sulphuret of carbon, be too large, both fall together to the bottom of the vessel. Here the camphor is mantled and dissolved by the sulphuret, and the instant the liquid spherule is raised to the surface of the water, it darts a film of camphor around it, and discovers uneven ridges throughout."—*J. Murray.*

11. *Dark-brown Streak on the Sea occasioned by Crabs.*

On the 6th December 1815, Captain Kotzebue observed on the surface of the sea, near the Island of St. Catherine, a serpentine streak, about two fathoms broad, of a dark brown colour, which extended as far as the eye could reach. Upon examining it, it was found to be occasioned by an innumerable quantity of small crabs, and the seeds of a marine plant.—*Kotzebue's Voyage of Discovery*, Vol. I. p. 113.

12. *Use of Phosphoric Acid in Jaundice.*

Dr. CALEB MILLER, of Bristol, (U. S.) has employed with considerable success the phosphoric acid, internally, for the cure of jaundice. He directs a large table spoonful of the acid, prepared according to the directions contained in *Murray's Materia Medica*, to be added to a pint of balm tea, and the mixture to be taken as fast as the stomach will bear it, till it operates as a diuretic. In a very obstinate case of jaundice, which was ultimately cured by this remedy, Dr. Miller states, that the patient took eight pints of the mixture in four-and-twenty hours.

13. *Chemical Analysis of the Humour of Porrigo.*

M. MORRIN, a chemist at Rouen, has been lately engaged in ascertaining the composition of the humour generated in this cutaneous disease. He found it to contain the following substances:—1. Ammonia in the state of an acetate, with excess of acid: 2. Osmazome; 3. Gelatine; 4. Fluid albumen, in very small quantity; 5. Concrete albumen, very abundant; 6. A fatty substance; 7. The chlorate of sodium; 8. Traces of the phosphate and of the sulphate of lime.—*Nov. Journ. de Med.* p. 239, vol. x.

14. *Analysis of Black and Green Tea.*

The following table is given by Mr. BRANDE as showing the respective quantities of soluble matter in water and alcohol, the weight of the precipitate by isinglass, and the proportion of inert woody fibre in green and black tea of various prices:—

| One hundred parts of tea. | Soluble in water. | Soluble in alcohol. | Precipitate with jelly. | Inert residue. |
|---|---|---|---|---|
| Green hyson, 14s. per lb... | 41 | 44 | 31 | 56 |
| Ditto, 12s. | 34 | 43 | 29 | 57 |
| Ditto, 10s. | 36 | 43 | 26 | 57 |
| Ditto, 8s. | 36 | 42 | 25 | 58 |
| Ditto, 7s. | 31 | 41 | 24 | 59 |
| Black souchong, 12s.... | 35 | 36 | 28 | 64 |
| Ditto, 10s. | 34 | 37 | 28 | 63 |
| Ditto, 7s. | 36 | 35 | 24 | 64 |
| Ditto, 6s. | 35 | 31 | 23 | 65 |

*Royal Institution Journal.*

☞ Dr. GRANVILLE was unanimously elected on the 19th of March, physician accoucheur to the Benevolent Institution.

### METEOROLOGICAL JOURNAL.

By Messrs. WILLIAM HARRIS and Co. 50, Holborn, London.

*From February* 20, 1822, *to March* 19, 1822.

| | MOON. | Rain gauge | THERM. | | | BAROM. | | DE LUC'S HYG. | | WIND. | | ATMOSPHERIC VARIATION. | | |
|---|---|---|---|---|---|---|---|---|---|---|---|---|---|---|
| | | | 9 A.M. | MAX. | MIN. | 9 A.M. | 10P.M. | 9 A.M. | 10P.M. | 9 A.M. | 10P.M. | 9 A.M. | 2 P.M. | 10P.M |
| Feb. | | | | | | | | | | | | | | |
| 20 | | .10 | 43 | 49 | 36 | 30.08 | 30.10 | 61 | 60 | W var. | NNE | Cloudy | Rain | Fine |
| 21 | ● | | 38 | 46 | 35 | 30.37 | 30.46 | 63 | 55 | N | N | Fine | | |
| 22 | | | 37 | 49 | 37 | 30 36 | 30.22 | 61 | 59 | WNW | W | Cloudy | Fine | |
| 23 | | | 40 | 50 | 40 | 30.11 | 30.19 | 58 | 55 | NW | WNW | Cloudy | | |
| 24 | | | 43 | 52 | 48 | 30.16 | 30.21 | 59 | 61 | W | WNW | Fine | | Cloud. |
| 25 | | | 50 | 53 | 45 | 30.25 | 30.23 | 61 | 60 | WNW | W | Cloudy | | |
| 26 | | | 47 | 51 | 39 | 30.11 | 30.20 | 60 | 62 | W | N | Fine | | |
| 27 | | | 42 | 46 | 34 | 30.50 | 30.60 | 56 | 50 | NNW | NW | Fair | Fine | |
| 28 | ☾ | | 37 | 43 | 32 | 30.68 | 30.42 | 51 | 56 | N | WNW | Foggy | Fine. | |
| Mar | | | | | | | | | | | | | | |
| 1 | | | 35 | 47 | 39 | 30.29 | 30.26 | 55 | 60 | S | WSW | Foggy | Fine | Fair |
| 2 | | | 44 | 52 | 42 | 30.21 | 30.32 | 61 | 60 | SW | SW | Cloudy | Fine | Fair |
| 3 | | | 48 | 53 | 43 | 30.30 | 30.22 | 65 | 67 | SW | SW | VeryFine | | |
| 4 | | .03 | 47 | 52 | 41 | 30.11 | 29.97 | 62 | 62 | SW | SW | Foggy | Fine | Show'r |
| 5 | | | 43 | 53 | 40 | 30.01 | 29.86 | 74 | 67 | SW | SW | Foggy | Fine | Fair |
| 6 | | .25 | 42 | 50 | 39 | 29.65 | 29.62 | 65 | 67 | WNW | W | Rain | Sho'ry | |
| 7 | ○ | .28 | 42 | 51 | 40 | 29.52 | 29.66 | 63 | 65 | W var. | W | Cloudy | Rain | Fine |
| 8 | | .10 | 41 | 53 | 39 | 29.57 | 29.51 | 62 | 59 | WNW | W | Showery | Rain | Fine |
| 9 | | .15 | 41 | 52 | 41 | 29.68 | 29.74 | 65 | 60 | WNW | W | Showery | Rain | Fair |
| 10 | | .16 | 43 | 51 | 39 | 29.57 | 29.73 | 63 | 62 | WNW | NW | Showery | | Rain |
| 11 | | | 42 | 47 | 36 | 29.81 | 30.15 | 59 | 54 | NW | NW | Fine | Sho'ry | Fine |
| 12 | | | 40 | 45 | 40 | 30.24 | 30.38 | 55 | 55 | SW | SW | Foggy | Fair | |
| 13 | | | 44 | 53 | 43 | 30.43 | 30.35 | 51 | 50 | SW | S | Fine | | |
| 14 | | .03 | 47 | 55 | 41 | 30.20 | 30.15 | 60 | 62 | SSW | NE | Fine | Sho'ry | |
| 15 | ☽ | | 43 | 54 | 40 | 30.19 | 30.24 | 60 | 63 | W | SW | Foggy | Fair | |
| 16 | | | 45 | 56 | 48 | 30.20 | 30.15 | 65 | 71 | SW | WSW | Foggy | Sho'ry | |
| 17 | | .05 | 50 | 57 | 49 | 30.12 | 30.04 | 68 | 72 | WSW | WSW | Fair | Sho'ry | Fair |
| 18 | | | 50 | 53 | 48 | 30.33 | 30.33 | 62 | 61 | N | WNW | Fine | Cloud. | Fair |
| 19 | | | 53 | 60 | 51 | 30.28 | 30.30 | 62 | 60 | WNW | W var. | Fine | Cloud. | Fair |

The quantity of rain during the month of February, was 0.93 of an inch.

